# Proposal-Based Visual Tracking Using Spatial Cascaded Transformed Region Proposal Network

**DOI:** 10.3390/s20174810

**Published:** 2020-08-26

**Authors:** Ximing Zhang, Shujuan Luo, Xuewu Fan

**Affiliations:** 1 Faculty of Space, Xi’an Institute of Optics and Precision Mechanics of CAS, Xi’an 710119, China; fanxuewu@opt.ac.cn; 2 School of Astronautics, Northwestern Polytechnical Universty, Xi’an 710072, China; luoshujuan@nwpu.edu.cn

**Keywords:** visual tracking, spatial cascaded networks, shrinkage loss, multi-cue proposals re-ranking, region proposals networks

## Abstract

Region proposal network (RPN) based trackers employ the classification and regression block to generate the proposals, the proposal that contains the highest similarity score is formulated to be the groundtruth candidate of next frame. However, region proposal network based trackers cannot make the best of the features from different convolutional layers, and the original loss function cannot alleviate the data imbalance issue of the training procedure. We propose the Spatial Cascaded Transformed RPN to combine the RPN and STN (spatial transformer network) together, in order to successfully obtain the proposals of high quality, which can simultaneously improves the robustness. The STN can transfer the spatial transformed features though different stages, which extends the spatial representation capability of such networks handling complex scenarios such as scale variation and affine transformation. We break the restriction though an easy samples penalization loss (shrinkage loss) instead of smooth L1 function. Moreover, we perform the multi-cue proposals re-ranking to guarantee the accuracy of the proposed tracker. We extensively prove the effectiveness of our proposed method on the ablation studies of the tracking datasets, which include OTB-2015 (Object Tracking Benchmark 2015), VOT-2018 (Visual Object Tracking 2018), LaSOT (Large Scale Single Object Tracking), TrackingNet (A Large-Scale Dataset and Benchmark for Object Tracking in the Wild) and UAV123 (UAV Tracking Dataset).

## 1. Introduction

Visual tracking has drawn constant attention of the researchers and engineers over last decades. Some novel applications are also inspired by the improvement of related research, such as auto-track by drone [1], pose recognition by mobile payment [2], and remote control by space robot [3]. Although the researchers are making much progress persistently, it is still a vital problem to achieve a tracking procedure that simultaneously balances the accuracy, robustness, and tracking speed under complex scenarios, such as occlusion, illumination change, and scale variation, to name a few [4].

Much progress [5] has made by the combined region proposal networks (RPN) and Siamese networks recently [6]. Some of the trackers treat the tracking problems as the generation of the similarity response map, which could distinguish the differences between the target templates and the search candidates. The position candidates where reach the highest similarity score is performed as the new target groundtruth. SiamRPN [7] combines Siamese networks and region proposal networks in order to jointly perform classification and regression for tracking. The DaSiamRPN [8] comes up with the distractor-aware module to distinguish hard negatives from easy ones, which could improve the discriminating power of such model. The other methods formulate the tracking problems as tracking-by-detection problems, which firstly generate the proposals from the search area, then calculate the classification score between proposal candidates and template target. The SiamRPN++ [9] introduces a ResNet-driven Siamese tracker, which makes layer-wise and depth-wise aggregations explicit when modeling network architecture, which can improve the accuracy and reduce the model size at the same time. The DCFNet [10] further combines the Siamese network with region proposal networks, and performs the domain specific updating to achieve a light-weight network in end-to-end learning.

**Figure 1:**
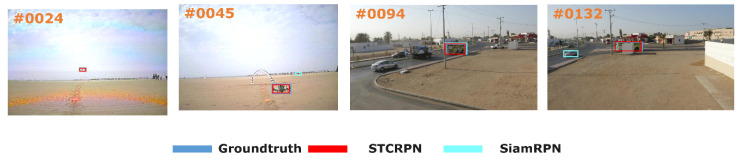
Comparison with the target groundtruth, SiamRPN and Spatial Cascaded Transformed Region Proposal Network (SCTRPN) on two sequences: It performs that the comparison between two different region proposal networks (RPN)-based methods in UAV123 dataset. The deep convolutional features can only be extracted to obtain the semantic information, it may easily drift away when suffering from the similar distractors and heavy occlusions.

There are still a few vital problems have not been settled during the tracking procedure though the region proposal networks based trackers introduced above have achieved excellent performance both in accuracy and speed. We notice that most trackers still employ the semantic features though the networks which cannot ensure the tracking accuracy which is shown in Figure 1. During target localization, One-stage region proposal network [11] proposes to perform a single regressor depends on pre-defined anchor boxes, while it is still difficult to estimate the changing scale of target [12]. The RPN-based trackers applies the log loss function as classification loss function which cannot effectively reduce the easy samples though training stage. Since the proposals generated by the proposed networks need to be strictly screened, the more effective proposals re-ranking method need to be proposed.

To overcome the restriction, we introduce a simple spatial cascaded strategy to apply the different hierarchical features from convolutional layers. By analyzing the feature transfer function of the spatial transformer networks (SPN), we successfully perform it as feature extraction model. The localization network of STN can obtain the position of target in image frame accurately, which helps us to solve the spatial transformation problems when suffering from heavy scale change and rotation. Benefiting from the shrinkage loss, we could penalize the weights of easy samples to alleviate the data imbalance issue. Considering the redundancy of the proposals, we find that multi-cue such as shape, color, and scale can be applied to refine the high-quality proposals that can not only improve the tracking performance in complex scenarios, but also reduce the computational effort. We name our proposed method as SCTRPN (Spatial Cascaded Transformed Region Proposal Network).

To summarize, the main contributions of this work are threefold and are listed below:

1. We present the spatial cascaded region proposals networks that combine region proposal networks and spatial transformer networks. In this circumstance, the deep and shallow layers’ features can be extracted to represent the appearance and semantic characteristic of a certain target.

2. We propose to employ the shrinkage loss to penalize the weighting coefficient of easy samples during the training procedure of the proposed networks to successfully reduce the samples imbalance issue.

3. We provide the multi-cue proposals re-ranking method for the tracking frameworks, which helps to refine the high quality proposals from the candidates.

The rest of the paper is organized as follows: in Section 2, we illustrate the related research work of our proposed method. In Section 3, we describe the proposed tracking framework in details, including network structure, feature extraction model, loss function design, and a proposals ranking strategy. In Section 4, we perform the numerous experimental results on the tracking dataset. In Section 5, we summarize the conclusions drawn from our proposed method.

## 2. Related Work

Visual tracking based on deep convolutional networks have shown significant potentials in recent decades. In the following section, we discuss the most relevant work, and refer readers to [13,14] for recent surveys.

### 2.1. Deep Tracking

At their very beginning, deep neural networks were introduced to deal with the image recognition problem [15]. Inspired by its successes, researchers are paying more attention to the application of CNN frameworks in visual tracking. Wang et al. [16] proposed to employ the fully convolutional network to accomplish a tracking procedure with an improved accuracy of estimated bounding box. Danelljan et al. [17] proposed a continuous convolution operator to combine the discrete features with the deep features, and achieve the efficient integration of deep feature maps by training spatial continuous convolution filters. Danelljan et al. [18] also proposed a factorized convolution operation to obtain an efficient convolution operator (ECO) for visual tracking, in order to prevent the low efficiency caused by the convolutional neural network. Song et al. [19] performed different kinds of adversarial networks to generate variable samples, which helped to identify richer representation for tracking. Fiaz et al. [20] proposed a soft mask feature fusion mechanism, which can be easily integrated into the conventional Siamese tracking framework to enhance the discriminative capability when distinguish the target from the background. Gordon et al. [21] introduced the real-time recurrent regression networks to combine the multiple appearance features and motion information together, then perform the spatial-temporal fusion to accomplish a tracking network that increases the precision of the tracking results. Guo et al. [22] proposed an effective online update mechanism using the dynamic Siamese matching strategy, and the FFT (Fast Fourier Transform) acceleration can ensure the real-time processing.

### 2.2. Tracking through Region Proposal Networks

Region proposal networks (RPN) draw much attention as an effective approach when faced with target detection problems. They also have increasing potential for visual tracking for its object classification and bounding box regression function. [23].

As far as we known, Li et al. [7] first interpolated the region proposal network to the Siamese network, and obtained the one-stage Siamese-RPN tracker to gain tracking performance. Zhu et al. [8] introduced the utilization of much more negative samples to train a distractor-aware Siamese-RPN tracker. Despite the fact that the distractor-aware tracker has achieved a significant improvement, it requires large extra training data from other computer vision datasets.

### 2.3. Tracking though Multiple Features Fusion

The multi-layer features strategy had made outstanding progress through the visual tracking procedure. The features from shallow layers contains more spatial information [24]. On the other hand, the deep layer features represent more semantic cues. Benefitting from the multi-features, tracking can perform the balanced spatial accuracy and robustness. In order to learn multiple correlation filters, Ma et al. [25] extracted the hierarchical convolutional features from three different layers of both deep and shallow networks. Wang et al. [16] proposed to employ two regression models, which contain the features from hierarchical convolutional layers to distinguish similar distractors.

### 2.4. Loss Function Variation for Data Imbalance

The loss function acts an important role in deep convolutional tracker by solving the data imbalance problem [26], though little attention had been paid to this kind of issue [27]. So far, the cost-sensitive loss [28] is proven to be an effective approach when suffering data imbalance. When pre-training the Siamese networks, Bertinetto et al. [29] proposed to balance the loss of positives and negatives in order to improve the discriminative ability of the network. Li et al. [30] used a temporal sampling scheme to balance positive and negative samples to facilitate CNN training.

### 2.5. Our Approach

**Figure 2:**
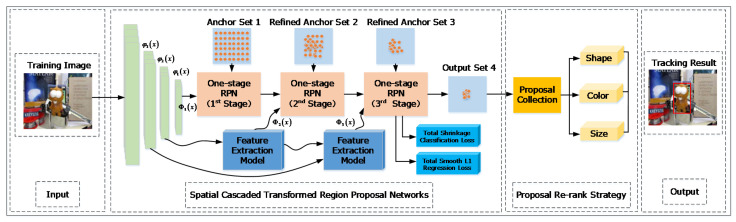
Flow diagram of the proposed tracking algorithm. The improvements which should be noticed are the feature extraction model, shrinkage classification loss, and proposal re-ranking strategy.

In this paper, we introduce the multi-stage spatial cascaded region proposal networks to generate the high-quality proposals. The baseline of our proposed method is one-stage region proposal network, which does not take data imbalance problem into consideration. On the contrary, our method proposes the spatial cascaded frameworks mainly to address the problem and filter the easy samples. We also introduce spatial transformer networks (STN) to perform the feature extraction and transfer procedure, which helps to improve the spatial transformer robustness. The shrinkage loss is also utilized to constrain the weights of easy samples during the training procedure. In addition, the multi-cue proposal re-ranking method is proposed to effectively refine the proposal candidates, improving the capability of maintaining the hard samples, which can distinguish the target from complex scenario. We name our proposed method SCTRPN. Figure 2 shows the flow diagram of our proposed method.

## 3. Proposed Method

### 3.1. Spatial Cascaded Region Proposal Networks

#### 3.1.1. One-Stage Region Proposal Network

**Figure 3:**
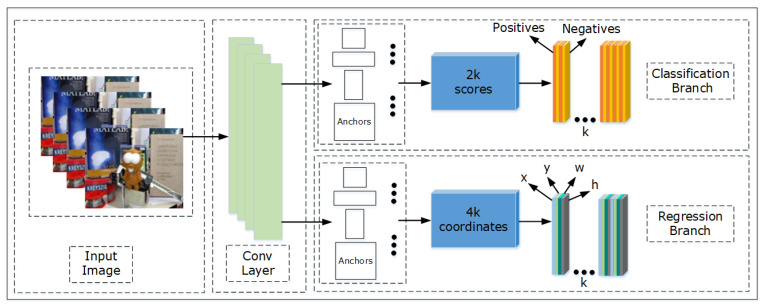
A brief structure of one-stage region proposal network in our networks. The main structure of one-stage region proposal network contains two branches, including classification and regression, which help the network seizing the proposals for detection or tracking.

Before describing SCTRPN, we first represent the one-stage region proposal network [23], including both classification and regression branches. The anchors are be obtained by the network structure, which is shown in Figure 3.

For training the one-stage region proposal network, we first assign to each anchor the binary class label, including the positives and negatives. The positives contain the anchors that has an IoU (intersection-over-union) overlap higher than 0.7 with any groundtruth bounding box. The negatives represent the anchors that has an IoU lower than 0.3 with the groundtruth. The positives and negatives are applied to the training objective.

During the training procedure, we can obtain the classification scores $\left\{p_{i}\right\}$ and the regression offsets $\left\{t_{i}\right\}$ for each anchor by computing $L\left(\left\{p_{i}\right\},\left\{t_{i}\right\}\right)$. We minimize the loss function based on the multi-task loss in Fast R-CNN [31]. Thus, the loss function for one-stage region proposal network is defined as,

(1) $$L\left(\left\{p_{i}\right\},\left\{t_{i}\right\}\right)=\frac{1}{N_{cls}}\sum_{i}L_{cls}\left(p_{i},p_{i}^{*}\right)+\lambda \frac{1}{N_{reg}}\sum_{i}p_{i}^{*}L_{reg}\left(t_{i},t_{i}^{*}\right),$$

where i represents the index of the anchor, and $p_{i}$ is the probability of anchor i, which represents the object. When the anchor belongs to the positives, the label $p_{i}^{*}$ is assigned to 1. The anchor is negative if the label is 0. $t_{i}$ represents the 4 coordinates of the predicted bounding box, and $t_{i}^{*}$ is the groundtruth box, which is affiliated with the positive anchor. The classification loss $L_{cls}$ is log loss between the object and non-object. The regression loss is represented by $L_{reg}\left(t_{i},t_{i}^{*}\right)=R\left(t_{i}-t_{i}^{*}\right)$, where R represents the smooth $L_{1}$ loss function which is defined in [32]. The term $p_{i}^{*}L_{reg}$ means that the regression loss can be activated when $p_{i}^{*}$ equals to 1, and is disabled when $p_{i}^{*}$ equals to 0. The outputs of the cls and reg layers consist of $\left\{p_{i}\right\}$ and $\left\{t_{i}\right\}$ respectively. The one-stage region proposal network is illustrated in detail in [20]. It can be employed to obtain the proposals for a visual tracker. When obtaining the proposals, we can perform the tracking procedure by calculating the maximum similarity score from the proposal candidates and target. However, the proposals that were obtained by the one-stage region proposal network usually cannot meet the requirements of trackers due to its low recall, which makes the tracker drift from the complex scenarios.

#### 3.1.2. The Proposed Networks

The previous RPN-based trackers only employ the high-level semantic features from the last layer, which leads to the class imbalance. The phenomenon may result in unpromising performance when suffering from similar distractors. Faced with these problems, we introduce the multi-stage tracking framework, which is able to combine a set of L(L≤N) RPNs for the proposals generation.

For the $l^{th}\left(1<l\leq L\right)$ stage $RPN_{l}$, it receives fused features $\Phi_{l}\left(x\right)$ of the conv-l layer and the high-level layers from feature extraction model (FEM), instead of features $\varphi_{l}\left(x\right)$ from a single separate layer [7]. The $\Phi_{l}\left(x\right)$ are obtained as follows,

(2) $$\Phi_{l}\left(x\right)=FEM\left(\Phi_{l-1}\left(x\right),\varphi_{l}\left(x\right)\right),$$

where denotes the FEM(⋅,⋅), as described in Section 3.2. For $RPN_{1}$, $\Phi_{1}\left(x\right)=\varphi_{1}\left(x\right)$. We can obtain the related classification scores $\left\{p_{i}^{l}\right\}$ and regression offsets $\left\{t_{i}^{l}\right\}$ for the specific anchors in stage l as follows,

(3) $$\begin{array}{l} \left\{p_{i}^{l}\right\}=L_{cls}^{shr}\left(\Phi_{l}\left(x\right)\right)\\ \left\{t_{i}^{l}\right\}=L_{reg}\left(\Phi_{l}\left(x\right)\right), \end{array}$$

where $L_{cls}^{shr}\left(\Phi_{l}\left(x\right)\right)$ classification loss function $L_{cls}$ (shrinkage loss), which is illustrated in Section 3.3 in detail, and $L_{reg}\left(\Phi_{l}\left(x\right)\right)$ are achieved by accomplishing the convolutional operations on $\Phi_{l}\left(x\right)$.

**Figure 4:**
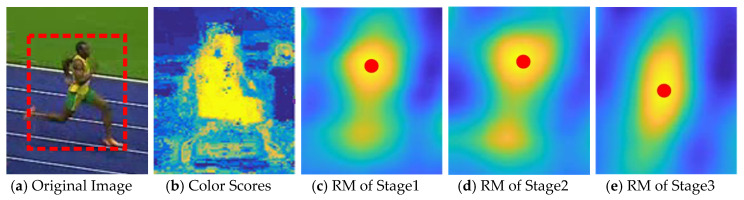
The comparison between the original image, color scores and different-stage response maps. (**a**) Contains the original image and the region of interest, (**b**) illustrates the color scores, and (**c**–**e**) clearly provide the multi-stage response maps.

Assuming that $A_{l}$ represents the anchor set in stage l. The negative anchors $A_{l}$ can be filtered out by threshold θ according to the classification scores $\left\{p_{i}^{l}\right\}$, we screen the anchors whose confidences are larger than the pre-defined threshold. We then achieve the positive anchors into a new set of anchors $A_{l+1}$. The positives are mainly utilized to train the networks. The initialization of the regression branch has a great influence on accurate anchor localization. In our method, we obtain the refined anchors $A_{l+1}$ by the the regression results $\left\{t_{i}^{l}\right\}$. Compared with the one-stage regression [23,25], the cascaded structure improved the accurate localization when transferring between different stages, as illustrated in Figure 4. We can see from Figure 4 that the results achieved by the response map of deep stage are closer to the center of the tracking target.

The loss function $\ell_{RPN_{l}}$ for $RPN_{l}$ is composed of classification loss function $L_{cls}^{shr}$ (shrinkage loss) and regression loss function $L_{loc}$ (smooth $L_{1}$ loss), which is shown in Equation (4),

(4) $$\ell_{RPN_{l}}\left(\left\{p_{i}^{l}\right\},\left\{t_{i}^{l}\right\}\right)=\sum_{i}L_{cls}^{shr}\left(p_{i}^{l},p_{i}^{l*}\right)+\xi \sum_{i}p_{i}^{l*}L_{loc}\left(t_{i}^{l},t_{i}^{l*}\right),$$

where i is the anchor index in $A_{l}$ of stage l, ξ is proposed to balance the classification and regression loss. By default, we set ξ=1, and thus, both $L_{cls}^{shr}$ and $L_{loc}$ are roughly equally weighted. We show by experiments that the training results are insensitive to the values of ξ. $p_{i}^{l*}$ represents the groundtruth label of anchor i, and $t_{i}^{l*}$ represents the distance between anchor i and groundtruth. Following [23], $t_{i}^{l*}=\left(t_{i\left(x\right)}^{l*},t_{i\left(y\right)}^{l*},t_{i\left(w\right)}^{l*},t_{i\left(h\right)}^{l*}\right)$ is a 4d vector, such that

(5) $$\begin{array}{l} t_{i\left(x\right)}^{l*}=\left(x^{*}-x_{a}^{l}\right)/w_{a}^{l}\;\;\;\;t_{i\left(y\right)}^{l*}=\left(y^{*}-y_{a}^{l}\right)/h_{a}^{l}\\ t_{i\left(w\right)}^{l*}=\log\left(w^{*}/w_{a}^{l}\right)\;\;\;t_{i\left(h\right)}^{l*}=\log\left(y^{*}/h_{a}^{l}\right), \end{array}$$

where x, y, w, and h represent the center of the tracking bounding box and its width and height, respectively. $x^{*}$ and $x_{a}^{l}$ are for the groundtruth and anchor of stage l (likewise for y, w and h). As far as we know, the previous method [7] proposed to utilize fixe anchors in RPN-based tracker. We employ the adjustable anchors in SCTRPN, which can change according to the regressor in the previous stage constantly, and computed as

(6) $$\begin{array}{l} x_{a}^{l}=x_{a}^{l}+w_{a}^{l-1}t_{i\left(x\right)}^{l-1}\;\;\;\;y_{a}^{l}=y_{a}^{l}+h_{a}^{l-1}t_{i\left(y\right)}^{l-1}\\ w_{a}^{l}=w_{a}^{l-1}\exp\left(t_{i\left(w\right)}^{l-1}\right)\;\;\;h_{a}^{l}=h_{a}^{l-1}\exp\left(t_{i\left(h\right)}^{l-1}\right), \end{array}$$

For the anchor in the first stage, $x_{a}^{1}$, $y_{a}^{1}$, $w_{a}^{1}$, and $h_{a}^{1}$ are predefined.

The proposed cascaded structure of the RPN module is formed above. We perform the easy negative anchors penalization, to ensure the balanced distribution of training samples gradually. The cascaded structure makes full use of the multi-level features, which make the classifier more discriminative in distinguishing intricate distractors in complex scenarios. Figure 4 also shows the discriminative powers of different RPNs by demonstrating the detection response map at each stage. The red spot represents the localization that achieve the highest response score.

The loss function $\ell_{SCTRPN}$ of SCTRPN consists of the loss functions of all $RPN_{l}$. We compute the isolated loss function by Equation (4), and $\ell_{SCTRPN}$ is expresses as

(7) $$\ell_{SCTRPN}=\sum_{l=1}^{L}\ell_{RPN_{l}},$$

### 3.2. Feature Extraction Model(FEM) though Spatial Transformer Network (STN)

Inspired by the affine robustness of the spatial transformer network (STN) proposed in [9], the feature extraction model is built upon a combination with STN. The STN is utilized to calculate the affine transformed parameters in order to make the classification procedure better. In [9], the STN helps to change the posture of object to meet the requirement of accurate classification. In our research, the STN acts as the feature extraction model to transfer the features of different stages to obtain multi-features. In addition, the original function can also make the features extracted more robust to target deformation.

**The Overview of STN.** The STN [32] consists of three essential parts, including localization network, grid generator and sampler. Given the feature map, we perform the localization network to estimate the translation, rotation, and scale of certain object. The variables for deformation will transfer to the grid generator for updated feature map grid generation, and the sampler can utilize the updated feature map to gain the deformed feature mapping. The deformed feature map can be transferred to next layer, in order to improve the affine robustness of trained networks. To be noticed, the STN is independent and can be inserted to any existing networks. The structure of STN in the existing networks can also be series and parallel. The whole process is differentiable when inserting the STN into the main networks. We can optimize the localization network, in order to gain the minimum classification objective though back-propagation directly.

**Figure 5:**
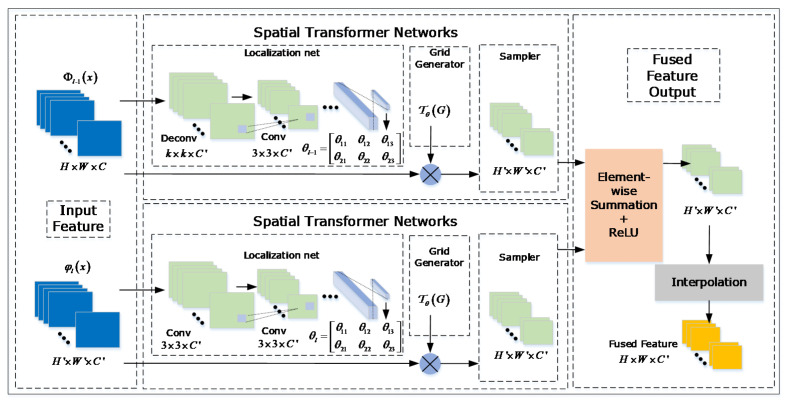
Flow chart of the feature extraction model though the spatial transformer network (STN). We employ the STN in order to transfer features to next stage and ensure dimension of feature simultaneously.

**Feature Extraction Model.** We obtain multi-level features from the multi-stages RPN structure, in order to effectively leverage these features, we propose to elect FEM to combine features across layers. During the combination, the high-level semantic features can be fused to improve the discriminability. In detail, the STN layer is used to transfer the feature map and match the feature dimensions at the same time. Different level features are fused by element-wise summation, followed a ReLU layer. We apply the grid generation model of STN to rescale the fused features, so that the FEM can ensure the same groundtruth for anchors in each RPN. In the meantime, we obtain the same resolution for all RPN in the output classification maps and regression maps. Figure 5 shows the feature extraction model for next layer.

In our experiments, we find it very important to limit the rotation degrees produced by FEM. Otherwise it is very easy to rotate the object upside down, which is the hardest to recognize in most cases. We constrain the rotation degree within 10 degrees clockwise and anti-clockwise.

### 3.3. Learning with Shrinkage Loss

Considering of the classification loss of the SCTRPN, we realize that the surrounding background contains much contextual information in. We can strengthen the discriminative power of classifier by utilizing the related wide background. In the meantime, it also brings large number of easy samples from the background, which cause the data imbalance issue. The easy samples may lead to undesirable results that generate the large loss. The learning process may pay much attention to the invaluable samples, which are far from the tracking target.

The research work in [33] found that the modulating factor can be applied to the loss, which can alleviate the data imbalance issue effectively. We treat the modulating factor as the function of the output possibility, and its function is to constraint the loss from easy samples.

Inspired by the shrinkage estimator [34] and the cost-sensitive weighting strategy [29], we propose the modulating factor, which is represented by l to re-weight the loss. We penalize the easy samples to achieve the hard samples by the optimization process. We compute the modulating function as a Sigmoid-like function by,

(8) $$f\left(l\right)=\frac{1}{1+\exp\left(a\cdot \left(c-l\right)\right)},$$

where a and c are hyper-parameters. The parameters are obtain to control the shrinkage speed and the localization, respectively. We apply the modulating factor to weight the log loss, the proposed shrinkage loss can be expressed as,

(9) $$L_{S}=\frac{l}{1+\exp\left(a\cdot \left(c-l\right)\right)},$$

The proposed shrinkage loss only penalizes the importance of easy samples (when l<0.5) and keeps the loss of hard samples unchanged (when l>0.5). Instead, we replace the classification loss by the shrinkage loss $L_{cls}^{shr}\left(p_{i},p_{i}^{*}\right)=-\log{\left(p_{i}\right)}_{p_{i}^{*}}$, which is employed in Equation (4),

(10) $$L_{cls}^{shr}\left(p_{i},p_{i}^{*}\right)=\frac{-\log{\left(p_{i}\right)}_{p_{i}^{*}}}{1+\exp\left(a\cdot c\right)\cdot {\left[{\left(p_{i}\right)}_{p_{i}^{*}}\right]}^{a}},$$

Considering of the implementation details, we set the value of a to be 10, in order to shrink the weight function quickly. We also set the value of c to be 0.2, so that the localization is suitable for the distribution of l. We constraint the value of c ranging from 0 to 1. Extensive comparison with the other losses shows that the proposed shrinkage loss can improve the tracking accuracy and the training speed at the same time.

### 3.4. Proposals Ranking Strategy

As we know, the high recall proposals can be generated by the spatial cascaded region proposal networks, while it may bring any redundancy to the tracking frameworks. For this reason, we present a multi-cue proposals re-ranking method, to obtain fewer and better proposals with high recall, which can provide the top tracking candidates for evaluation. The re-ranking method is based on multiple cues between proposal candidates and groundtruth, which include shape, color, and scale, which is shown in Figure 2.

**Shape.** The contours which enclosed by the bounding box ρ can be calculated by the existing method [35]. Furthermore, we can treat the number of contours enclosed by bounding box as shape score. Thus, the shape cue value $s_{i,t}$ between the i-th proposal candidate and target region $\tau_{t}$ is illustrated by,

(11) $$s_{i,t}=e^{\left(-\left|\rho_{i}-\rho_{t}\right|\right)},$$

where $\rho_{i}$ and $\rho_{t}$ represent the shape score of i-th proposal and the target, respectively.

**Color.** The color $c_{i,t}$ between the i-th proposal candidate and the target can be computed by the response map. The mean of all the values of the pixels from the region of the response map corresponded to the i-th proposal candidate, is computed as the color value between the i-th proposal candidate and target.

**Size.** We propose to utilize the size information to filter out the mismatching proposal candidates, which are undersized or oversized. The size value $z_{i,t}$ between the i-th proposal candidate and target is defined as,

(12) $$z_{i,t}=e^{\left(-\left|\omega_{i}-\omega_{t}\right|\right)}\cdot e^{\left(-\left|h_{i}-h_{t}\right|\right)},$$

where $\omega_{i}/h_{i}$ and $\omega_{t}/h_{t}$ denote the width/height of the i-th object proposal candidate and the target, respectively.

The three cues introduced above are independent, thus the multi-cues $a_{i,t}$ between the i-th proposal candidate and target can be performed as the product of the three core cues by,

(13) $$a_{i,t}=s_{i,t}\cdot c_{i,t}\cdot z_{i,t},$$

During the proposals re-ranking process, we propose to rank the proposal candidates though the multi-cues in descending order. Then, we could obtain the high-quality proposals successfully by wiping out the proposal candidates with the low values. The high-quality proposals can maintain high recall, which helps the tracker to achieve better performance.

## 4. Experimental Results and Analysis

### 4.1. Training Dataset and Evaluation

**Training.** We train the SCTRPN by random interval sampling the images from the same sequences. Usually, we generate at most 64 samples from one image. We also perform the end-to-end network training, due to the combined spatial cascaded loss function. When it comes to the ratio of the anchor, the scale of target change smoothly between two consecutive frames in most situations, except for the fast motion sequences. Thus, we assign the ratios of anchors to [0.33,0.5,1,2,3] which is same as [7]. As for the positives and negatives, the positives are represented by the anchors, whose intersection-over-union (IOU) with groundtruth is over the threshold $\theta_{pos}$. On the contrary, the negatives are defined by the anchors, whose IOU with groundtruth is less than the threshold $\theta_{neg}$. The settings of shrinkage loss are described in Section 3.3.

**Tracking.** We evaluate the short-term object tracking on OTB2015 [36], VOT2018 [37], and UAV123 [1], respectively. LaSOT [38] and TrackingNet [39] are two recent largest datasets for single object tracking, and we validate the proposed method on these two datasets, to test its generalization performance.

The tracking procedure is more like the multi-stage detection: we first extract the features from the image in the first frame, using the pre-trained networks. For each stage, we utilize the FEM to combine the features and calculate the classification score and regression offset. Then, we perform coarse refining the anchor though RPN. The remaining anchors are regarded as proposal candidates, from which we utilize the multi-cues proposal re-ranking strategy to filter out the candidates in descending order, to obtain high-quality proposals. The final tracking results are determined by non-maximum-suppression (NMS), which is performed afterwards to get the final tracking bounding box. After the final bounding box is selected, the target size is updated by linear interpolation, to keep the shape changing smoothly. The whole tracking process of SCTRPN is summarized in Algorithm 1.
**Algorithm 1** Proposed Tracking Method. **Input:** Given sequences ${\left\{X_{t}\right\}}_{t=1}^{T}$; Groundtruth boundingbox of first frame $X_{1}$ named $b_{1}$; The trained model SCTRPN; **Output:** Tracking results ${\left\{b_{t}\right\}}_{t=2}^{T}$; Initialize anchors $A_{1}$;  **For**
t=2 to T do    Extract features ${\left\{\varphi_{l}\left(x\right)\right\}}_{l=1}^{L}$ for x from SCTRPN;    **For**
l=1 to L do    **If**
l equals to 1 **then**    $\Phi \left(x\right)=\varphi_{l}\left(x\right)$;    Else    $\Phi_{l}\left(x\right)=FEM\left(\Phi_{l-1}\left(x\right),\varphi_{l}\left(x\right)\right)$;    End    Calculate the classification score and regression offset using Equation (3);    Coarse refining the anchor i from $A_{l}$ using Equation (6);    Fine re-ranking the proposal candidates using multi-cues re-ranking strategy in Equation (13);  **End**    Select the best proposal as tracking result $b_{s}$ by the selection strategies in [22];  **End**

### 4.2. Implementation Details

**Network Architecture.** In experiments, the backbone networks adopts the AlexNet [15] by reserving Conv layers to extract the features of images. The networks framework is described in detail in Section 3.1. The networks we combined include AlexNet, spatial transformer networks, and region proposal networks.

**Optimization.** We implement the whole training and tracking process using MatConvNet Deep Learning Frameworks [40] on a PC with an Intel i7, 16GB RAM and single Nvidia GTX1080Ti with 11GB video memory. The pre-trained parameters are directly come from the existing model on ImageNet [15]. SCTRPN is end-to-end trained with stochastic gradient descent (SGD) by 40 epoches. We employ a warmup learning rate of 0.001 for first 5 epoches to train the RPN braches. For the last 15 epoches, the whole network is end-to-end trained with the learning rate exponentially decayed from 0.001 to 0.00001. We set the stage number L to 3. We also assign the IOU of the positives and negatives to 0.7 and 0.3, respectively. The training loss is illustrated in Equation (7).

### 4.3. Relablity Ablation Study

**Multi-features fusion through FEM.** In order to test the validation of the multi-stage feature combination though FEM, we first to do the experiments on VOT2018 dataset, and the results are shown in Table 1. We firstly test the one-stage tracker and obtain the competitive performance with 0.321 in EAO. When we add another stage to the baseline, the EAO has increased to 0.352, and the accuracy and robustness are both increased by 8% and 17%, respectively. After combining all three stages, both accuracy and robustness steadily improve, with gains between 2.1% and 4.9% for VOT2018, compared with two-stage results. When it comes to EAO, the three stages are 12.4% higher than that of a single stage. We also provide the experimental results on different stages without STN, which can be seen in the last three lines of Table 1. The Tracker with STN outperforms those without STN in three main metrics except the tracking speed due to the accurate localization and affine transformed evaluation of the proposed FEM based on STN. The spatial transformer networks in FEM makes the effective progress when the image sequence suffering from deformation changes and affine transformation which refers to the attributes-based comparison in Section 4.4. Considering of the tracking speed, the three-stage tracker can also meet the demand of real-time tracking, though it costs more computational time during the tracking process.

**Shrinkage loss.** When replacing the proposed shrinkage loss with the original log loss, we evaluate the experimental analysis on the VOT2018 dataset. Compared with the RPN baseline, the proposed loss gains the large margin of 0.361, 5.1% higher than log loss. We also compare our proposed method with online hard negative mining [41], which aims to evaluate the capability of alleviating the data imbalance issue. Both methods penalize the importance of easy samples, due to the attribute of cost-sensitivity. We experimentally set the threshold of mining to 0.01. Our proposed method outperforms the online hard negative mining method. We can infer from the comparative results that easy samples still contribute to the learning process, but they should not dominate the whole gradient. Online hard negative mining was proposed to manually set the threshold, which cannot be appropriate for all the testing sequences.

**Figure 6:**
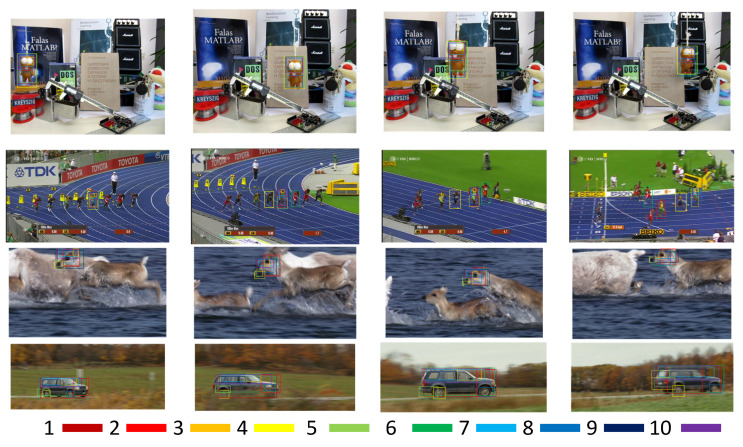
Qualitative proposal generation results obtained by the proposal re-ranking method on some videos of the OTB-2015 dataset. Only top ten generated proposals are shown in the test images.

**Multi-cue re-rank.** As shown in Figure 6, we evaluate the qualitative proposal generation results obtained by the proposal re-ranking method on some videos of the OTB-2015 dataset. Only the top ten generated proposals are shown in the test images. Under most circumstances, the top score generated proposals can cover the tracking groundtruth. We compare the proposed proposal re-ranking method with several state-of-the-art proposal generation methods in terms of recall. Four methods are chosen to achieve the evaluation, including CADM [42], MSTE [43], EdgeBoxes [44], and SelectiveSearch [45]. Selective Search and CADM are based on image segmentation; MSTE and EdgeBoxes are based on the boundary or the edge feature. The multi-cue re-ranking strategy has the capability of integrating the proposals around the target, and distinguishing the foreground target from the background clutters, which can obtain highly accurate proposal generation. As shown in Table 2, the recall obtained by multi-cues proposal re-ranking method is the highest among those obtained by the competing methods, higher by 34–70% when the top 50 ranked object proposals are used for calculating the recall. This is because the proposed method can combine color, shape, and scale information to accomplish the whole proposal generation process, which performs more robust to motion blur, illumination, deformation, and some other complex scenarios. In this section, we briefly test the proposal generation capability of our proposed networks. The OTB-2015 dataset, as a traditional benchmark, can clearly obtain the visualization of the results. Due to the eleven multiple tracking attributes, which almost contain all the tracking problems, the researchers still employ the OTB-2015 benchmark to accomplish a qualitative and quantitative comparison in recent paper work. We need to solve the out-of-view problems in the benchmark of OTB-2015 during proposals generation.

### 4.4. Comparison with State-of-the-Art Methods

**Figure 7:**
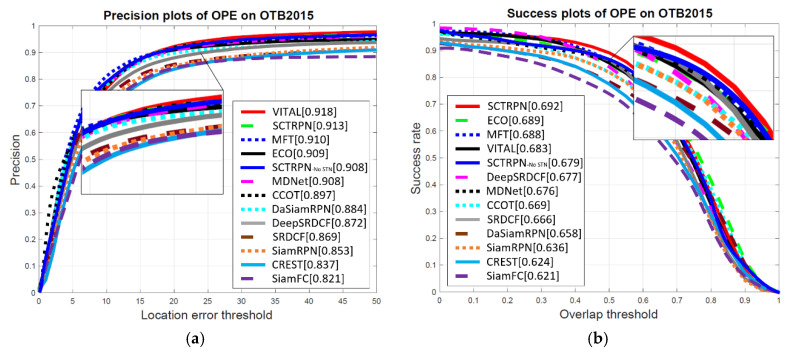
Precision and success plots show a comparison of our SCTRPN tracker with state-of-the-art trackers on the OTB2015 dataset. (**a**) Precision plots of OPE on OTB2015; (**b**) Success plots of OPE on OTB2015.

**OTB-2015 Dataset.** The OTB-2015 dataset contains 100 sequences that mainly evaluate the accuracy and robustness of the compared tracker via the location error ratio and overlap ratio. The RPN-based tracker formulate the tracking as one-shot detection without online updating during the whole procedure. As the proposed tracker employs the proposals to do the final tracking, it can overcome the fast motion attributes most times. The improved the classification loss and multi-cues proposals re-ranking also help the proposed tracker to be top-performing method. We compare our SCTRPN tracker on the OTB2015 with the state-of-the-art trackers. Figure 7 shows that our SCTRPN tracker produces the leading result in overlap success and ranks second place in precision plots. Compared with other RPN-based trackers, our SCTRPN outperforms DaSiamRPN by improving 3.2% in precision and 5.1% in success. The VITAL tracker, which is performed via adversarial learning representations, obtained the best results in precision, and the network has the capability of consistently amending the edge information though adversarial learning. To compare with the correlation filters method ECO [18], we elect the features fusion strategy for both, while the multi-stage features extracted by feature extraction model are less sensitive to deformation variation and illumination, which results in a more comparable performance. We should also notice that the online updating methods fail to outperform other methods in the precision plot, due to the drift away caused by template updating. We also evaluate the proposed tracking method without the STN model, the scores of the precision and success drop dramatically, according to the details in the figure.

**VOT-2018 Dataset.** We validate our SCTRPN tracker on the VOT-2018 dataset in comparison with seven other state-of-the-art methods. The VOT-2018 public dataset includes 60 public sequences with different challenging attributes. It is treated as one of the most recent datasets for evaluating online model-free single object trackers. According to protocol, the expected average overlap (EAO), accuracy (A), and robustness (R) and no-reset-based average overlap (AO) are used to compare different trackers. The comparisons between state-of-the-art methods are reported in Table 3, and red, blue represent 1st and 2nd, respectively. Table 3 shows that the proposed SCTRPN tracker achieves the top-ranked performance on EAO and AO criteria. We also rank second place in the criteria of accuracy. As for robustness, the MFT and LADCF rank 1st and 2nd place, respectively. Due to the multi-stage fusion and proposal re-ranking strategy, our proposed SCTRPN method yields substantial gains of 10.9% on accuracy. The STN plays the key role in feature extraction model. Without the STN model, the other RPN-based methods outperform the SCTRPN-No STN, due to the lack of appearance change processing. The SiamRPN also obtains a high accuracy, mainly considering of the Siamese matching structure. Compared with the LADCF method, the proposed method achieves a performance gain of 1.5% on EAO criteria. Due to not matching the template, the robustness still has a gap with the state-of-art Siamese based trackers. We adopt one pass evaluation to test the different trackers. From the results of the AO criteria, we can observe that our proposed method outperforms the SiamRPN and DaSiamRPN method by 3.4% and 8.9% on AO, that is to say, our method achieve comparable performance compared with other RPN-based methods, and also has the capability of long-term tracking.

**Figure 8:**
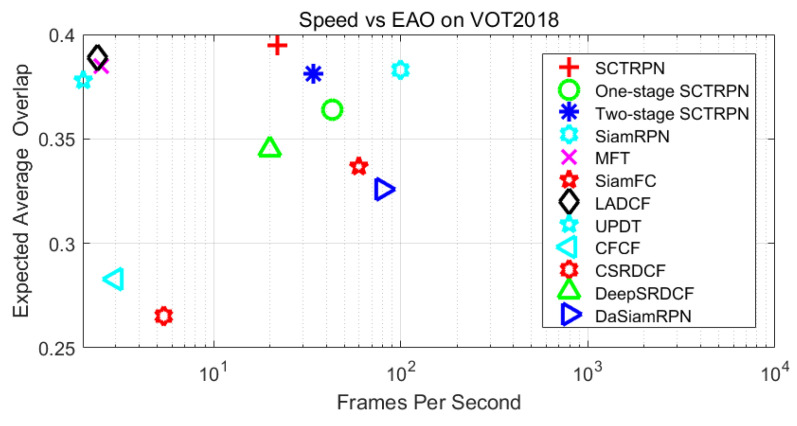
A comparison of the quality and the speed of state-of-the-art tracking methods on VOT2018. The expected average overlap (EAO) with respect to the frames-per-second (FPS) are visualized in the Figure. Note that the FPS axis is in the log scale.

**Accuracy vs. Speed.** As shown in Figure 8, we perform the EAO performance on VOT2018 dataset to evaluate the tracking speed with respect to frames-per-second (FPS). The proposed network is not that complex, thus the device we chosen is single NVidia GTX1080Ti with 16GB Memory. We compare the proposed method with the results provided by the VOT2018 official data. We visualize that our SCTRPN achieves the best performance compared with other state-of-the-art methods running at real-time speed (22 FPS). When it comes to the MFT and LADCF, the tracking speed is low, due to the computational complexity and hardware limitation. From Figure 8, we can see that the SiamRPN is the most efficient approach among the compared methods. However, the proposed method achieved the higher EAO score than that of the SiamRPN, and the tracking speed can also meet the demand of real-time processing (22 FPS) in the computer vision application. Compared with the one-stage and two-stage SCTRPN, the fewer stages we employ, the faster the trackers perform. We set the stage number L to 3, considering the balance between effectiveness and efficiency.

**Figure 9:**
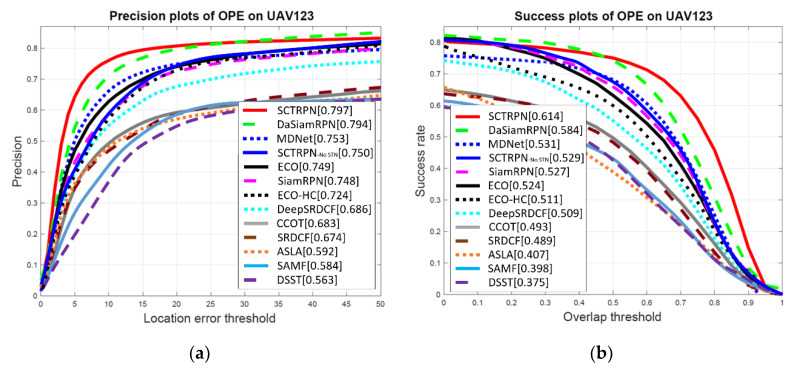
Normalized precision and success plots show a comparison of our SCTRPN tracker with state-of-the-art trackers on the LaSOT dataset. (**a**) Normalized Precision plots of OPE on LaSOT Testing Set; (**b**) success plots of OPE on the LaSOT Testing Set.

**LaSOT Dataset.** We elect the experiments on LaSOT to further validate the proposed method on a larger and more challenging dataset. We have got 1400 videos in total and 280 videos with large scale and high quality in the LaSOT dataset. Figure 9 reports the overall performances of our SCTRPN tracker on LaSOT testing set. Our SCTRPN method outperforms other state-of-the-art methods by 0.559 and 0.487 on normalized precision and success. Compared with the RPN-based method DaSiamRPN, our SCTRPN tracker increases the normalized distance precision and success by 12.7% and 18.2%. Due to the shrinkage loss, we could penalize the importance of easy samples to alleviate the data imbalance issue, which makes the tracker more appropriate for long-term tracking.

**TrackingNet Dataset.** The TrackingNet dataset contains a large amount of data in the wild, which can evaluate the trackers’ performance in complex scenarios. There are 511 videos in the dataset. Among these videos, the tracking object is mainly suffering from illumination variation, heavy occlusion and background clutters. The TrackingNet dataset provides three criteria including success (A), precision (P) and normalized precision (Pnorm) for evaluation. Table 4 visualizes the comparison results, the SCTRPN achieves the best results on all three criteria from the second row. Specifically, the SCTRPN achieves 69.7%, 66.4%, and 76.4% on A, P, and Pnorm, respectively. The RPN-based methods outperform the methods of other tracking frameworks. Among the RPN-based methods, DaSiamRPN tracker obtains the second best results of 63.8% and 59.2% on A and P, and the SiamRPN achieves the second best results of 74.1% on Pnorm. The SCTRPN tracker gains the capability of tracking the object in the wild, due to multi-stage feature fusion and proposals re-ranking strategy, thus, the proposed method is more appropriate to handle the attributes of illumination change, background clutters, and also scale variation.

**Figure 10:**
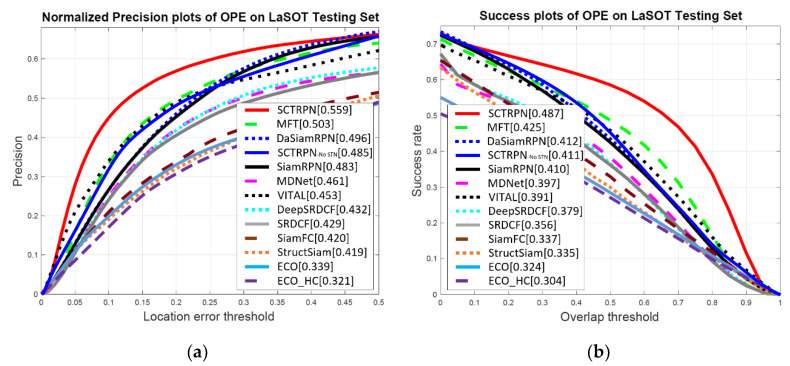
Precision and success plots show a comparison of our SCTRPN tracker with state-of-the-art trackers on the UAV123 dataset. (**a**) Precision plots of OPE on UAV123; (**b**) Success plots of OPE on UAV123.

**UAV123 Dataset.** UAV123 dataset includes 123 sequences with average sequence length of 915 frames, recent researches mainly employ the length of the UAV123 dataset to validate the long-term tracking ability of trackers. We test state-of-the-art methods on UAV123 dataset, and infer from the results that the convolutional features are more robust than conventional handcrafted features, and the networks-based tracking frameworks outperform other compared frameworks, such as correlation filters during long-term tracking. Figure 10 illustrates the precision and success plots of the compared trackers. Specifically, our tracker achieves 0.797 and 0.613 on precision and success ranking the first place, which outperforms other two RPN-based methods DaSiamRPN (0.794, 0.584) and SiamRPN (0.748, 0.527). As for ECO and ECO-HC, the ECO only gains the precision and success by 3.4% and 2.5%, compared with ECO-HC. The convolutional features do little to influence the filters based trackers in long-term tracking, while the multi-stage features fusion strengthen the ability of robust tracking in complex scenarios.

**Figure 11:**
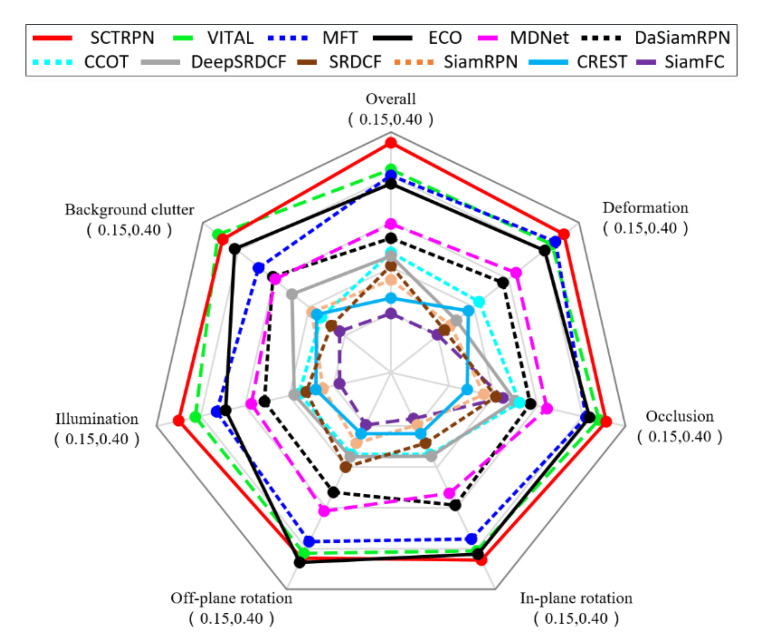
Attribute-based evaluation of the trackers. The experimental results show the validation of six attributes including deformation, occlusion, in-plane rotation, off-plane rotation, illumination change, and background clutter. The numbers under the name represent the range of the attributes respectively.

**Attribute-based Evaluation.** The VOT2018 dataset is per-frame annotated with visual attributes to allow the detailed analysis of per-attribute tracking performance. Six attributes are chosen to accomplish the attribute-based evaluation for their importance to the compared trackers, including deformation variation, heavy occlusion, in-plane rotation, off-plane rotation, illumination change, and background clutter. Figure 11 shows the per-attribute plot for the top-performing trackers on VOT2018 in EAO. The proposed method was consistently ranked among the top trackers on the four attributes. The proposed SCTRPN performs the best in terms of deformation variation, occlusion, in-plane rotation, and illumination change. In summary, our proposed SCTRPN method can handle the extreme tracking situation under complex scenarios.

### 4.5. Hyper Parameters Selection

**Different values of**ξ**.** In Table 5, we briefly test the different values of ξ in Equation (4). By default, we use ξ=1 which makes the two terms in Equation (4) roughly equally weighted after normalization. Table 5 shows that our result is impacted just marginally (by 2%) when ξ is within a scale of about two orders of magnitude (1 to 100). This demonstrates that the result is insensitive to ξ in a wide range.

**Figure 12:**
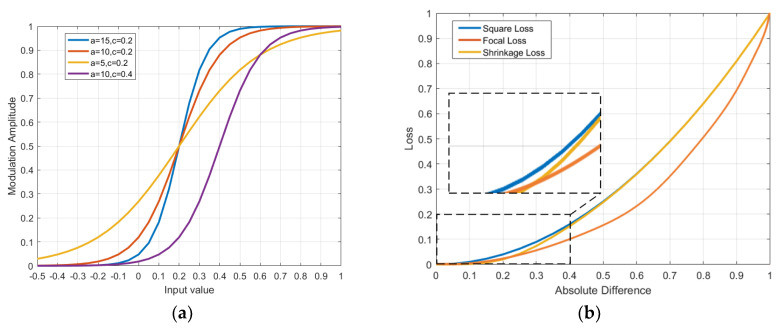
(**a**) Modulating factors in (5) with different hyper-parameters. (**b**) Comparison between the square loss (L2), focal loss (L3), and the proposed shrinkage loss.

**The values of a and c in shrinkage loss.** The modulating function is with the shape of a Sigmoid-like function, which is shown in Equation (8). Where a and c are hyper-parameters controlling the shrinkage speed and the localization, respectively. Figure 12a shows the shapes of the modulating function with different hyper-parameters. When applying the modulating factor to weight and the square loss, we have the proposed shrinkage loss as Equation (9). In this section, we mainly discuss the values of a and c hyper parameters in our proposed shrinkage loss. As shown in Figure 12b, the shrinkage loss only penalizes the importance of easy samples (when l < 0.5), and keeps the loss of hard samples unchanged (when l > 0.5) when compared to the square loss (L2). The focal loss (L3) penalizes both the easy and hard samples. We set the value of a to be 10 to shrink the weight function quickly and the value of c to be 0.2 to suit for the distribution of l, which ranges from 0 to 1. An extensive comparison with the other losses shows that the proposed shrinkage loss not only improves the tracking accuracy but also accelerates the training speed.

## 5. Conclusions

In this paper, we proposed a brand new framework for visual tracking which consists of spatial cascaded region proposals networks combining region proposal networks and spatial transformer networks to fully utilize multi-features. We also introduced the employment of the shrinkage loss to penalize the importance of easy samples to effectively alleviate the data imbalance issue. We provided the multi-cue proposals re-ranking method for the tracking frameworks, which can screen high quality proposals. Numerous experimental results demonstrated that the proposed tracker outperforms state-of-the-art trackers, highlighting the significant benefits of our method. However, we still cannot solve the problems of the “Tracking-by-understanding” mechanism. For future research, we will focus on the application of the combination between different networks accomplishing the action and pose detection during visual tracking. The enhancement of the deep learning network can not only improve the tracking performance, but also make the high-level computer vision applications based on tracking methods come true.

## Figures and Tables

**Table 1:** The reliability ablation study on multi-features fusion considering the number of stages and STN. The best two results are highlighted in **red** and **blue** fonts, respectively.

Stage	One Stage	Two Stages	Three Stages	One Stage without STN	Two Stages without STN	Three Stages without STN
**Accuracy** ↑	0.523	0.565	**0.577**	0.508	0.538	**0.566**
**Robustness** ↓	1.23	**1.02**	**0.97**	1.34	1.19	1.04
**EAO** ↑	0.321	**0.352**	**0.361**	0.314	0.342	0.349
**FPS** ↑	**45**	30	22	**54**	36	25

**Table 2:** The recall obtained by the proposal re-ranking method and the other four competing methods when varying the number of object proposals on the OTB-2015 dataset.

Methods	Number of Proposals				
	50	100	200	500	1000
CADM	0325	0.436	0.574	0.706	0.735
MSTE	0.253	0.424	0.567	0.632	0.653
EdgeBoxes	0.603	0.743	0.813	0.924	0.929
SelectiveSearch	0.246	0.392	0.521	0.732	0.841
SCTRPN	0.921	0.932	0.953	0.983	0.991

**Table 3:** Comparison with the state-of-the-art in terms of accuracy, robustness (failure rate), expected average overlap (EAO) and no-reset-based average overlap (AO) on the VOT2018 dataset. The best two results are highlighted in **red** and **blue** fonts, respectively.

Tracker	SCTRPN	SCTRPN-No STN	MFT	LADCF	DRT	SiamRPN	DaSiamRPN
**Accuracy** ↑	**0.583**	0.564	0.525	0.503	0.519	**0.586**	0.569
**Robustness** ↓	0.243	0.269	**0.140**	**0.159**	0.201	0.276	0.323
**EAO** ↑	**0.395**	0.381	0.385	**0.389**	0.357	0.382	0.327
**AO** ↑	**0.478**	0.453	0.393	0.421	0.426	**0.462**	0.439

**Table 4:** Comparison on the TrackingNet in terms of success, precision, and normalized precision. The best two results are highlighted in **red** and **blue** fonts, respectively.

Tracker	SCTRPN	SCTRPN-No STN	ECO	MDNet	SiamFC	SiamRPN	DaSiamRPN
**A(%)** ↑	**69.7**	62.7	55.4	60.6	57.2	62.4	**63.8**
**P(%)** ↑	**66.4**	59.4	49.3	56.8	53.6	58.4	**59.2**
**P_norm_(%)** ↑	**76.3**	73.9	62.1	71.2	66.6	**74.1**	73.2

**Table 5:** Tracking results of EAO on the VOT-2018 benchmark using different values of ξ in Equation (4). The best results are highlighted in **red** fonts.

ξ	ξ=0.1	ξ=1	ξ=10	ξ=100
**EAO** ↑	0.389	**0.395**	0.392	0.387
